# Efficacy of ChAdOx1 vaccines against SARS-CoV-2 Variants of Concern Beta, Delta and Omicron in the Syrian hamster model

**DOI:** 10.21203/rs.3.rs-1343927/v1

**Published:** 2022-02-15

**Authors:** Neeltje van Doremalen, Jonathan E. Schulz, Danielle R. Adney, Taylor A. Saturday, Robert J. Fischer, Claude Kwe Yinda, Nazia Thakur, Joseph Newman, Marta Ulaszewska, Sandra Belij-Rammerstorfer, Greg Saturday, Alexandra J. Spencer, Dalan Bailey, Sarah C. Gilbert, Teresa Lambe, Vincent J. Munster

**Affiliations:** 1 Laboratory of Virology, National Institute of Allergy and Infectious Diseases, National Institutes of Health, Hamilton, MT, USA; 2 Viral Glycoproteins Group, The Pirbright Institute, Pirbright, Woking, UK; 3 Pandemic Sciences Institute, Nuffield Department of Medicine, University of Oxford, Oxford, UK; 4 Rocky Mountain Veterinary Branch, National Institute of Allergy and Infectious Diseases, National Institutes of Health, Hamilton, MT, USA; 5 Oxford Vaccine Group, Department of Paediatrics, University of Oxford, Oxford, UK and Chinese Academy of Medical Science (CAMS) Oxford Institute (COI), University of Oxford, Oxford, UK

## Abstract

ChAdOx1 nCoV-19 (AZD1222) is a replication-deficient simian adenovirus–vectored vaccine encoding the spike (S) protein of SARS-CoV-2, based on the first published full-length sequence (Wuhan-1). AZD1222 was shown to have 74% vaccine efficacy (VE) against symptomatic disease in clinical trials and over 2.5 billion doses of vaccine have been released for worldwide use. However, SARS-CoV-2 continues to circulate and consequently, variants of concern (VoCs) have been detected, with substitutions in the S protein that are associated with a reduction in virus neutralizing antibody titer. Updating vaccines to include S proteins of VoCs may be beneficial over boosting with vaccines encoding the ancestral S protein, even though current real-world data is suggesting good efficacy against hospitalization and death following boosting with vaccines encoding the ancestral S protein. Using the Syrian hamster model, we evaluated the effect of a single dose of AZD2816, encoding the S protein of the Beta VoC, and efficacy of AZD1222/AZD2816 as a heterologous primary series against challenge with the Beta or Delta variant. We then investigated the efficacy of a single dose of AZD2816 or AZD1222 against the Omicron VoC. As seen previously, minimal to no viral sgRNA could be detected in lungs of vaccinated animals obtained at 5 days post inoculation, in contrast to lungs of control animals. Thus, these vaccination regimens are protective against the Beta, Delta, and Omicron VoCs in the hamster model.

## Introduction

At the end of 2019, the causative agent of COVID-19, severe acute respiratory syndrome coronavirus 2 (SARS-CoV-2), was first detected in Wuhan, China^1,2^. As of February 2^nd^ 2022, SARS-CoV-2 has infected an estimated 380 million people, causing more than 5 million deaths^3^. Its emergence prompted the rapid development of vaccines based on the viral receptor binding protein, spike (S)^4–6^. Several vaccines demonstrated efficacy through clinical trials in less than a year^7–11^ and were approved for emergency use by different regulatory bodies worldwide. Over 4.4 billion people are estimated to have received at least one dose of COVID-19 vaccination^3^. One of those vaccines is AZD1222 (ChAdOx1 nCoV-19), developed by Oxford University and produced by AstraZeneca. AZD1222 is a replication-deficient simian adenovirus–vectored vaccine encoding the non-stabilized S protein of Wuhan-1, one of the first published full-length SARS-CoV-2 sequences^12^. AZD1222 was shown to be highly effective in clinical trials, demonstrating 74% vaccine efficacy against symptomatic disease^7^. A two dose primary series of AZD1222 is approved for usage in more than 170 countries, and more than two billion doses of vaccine have been distributed worldwide^13^.

Despite the development and administration of these vaccines, a large portion of the world’s population is still unvaccinated, particularly in low-income countries^3^. Furthermore, although COVID-19 vaccination is protective against severe disease, it is not fully protective against infection with SARS-CoV-2, and breakthrough infections regularly occur^14^. High levels of circulating virus, asymptomatic infections, low vaccine coverage and break-through infection together means SARS-CoV-2 continues to circulate in the population. As a consequence, several variants of concern (VoCs) have been detected. A variant is termed a VoC if it is associated with an increase in transmission or virulence, or a decrease in the effectiveness of public health and social measures, such as diagnostics, vaccines, or therapeutics^15^. Since COVID-19 vaccines were developed early in the pandemic, they are based on the ancestral S protein, and substitutions in S may result in a reduced vaccine efficacy against VoCs. Several VoCs have substitutions in the receptor binding domain of S which are associated with a reduction in neutralizing virus titers^16–20^, which is a strong predictor of vaccine efficacy^21^, but the current vaccines clinically available are largely able to protect against severe disease and hospitalization caused by VoCs^22,23^. Here, we investigate the efficacy of an updated vaccine based on the S protein of the Beta variant (AZD2816)^24^ against three different VoCs; the Beta, Delta, and Omicron variants, relative to the original AZD1222 vaccine.

## Results

**Extended Data Figure 1.**
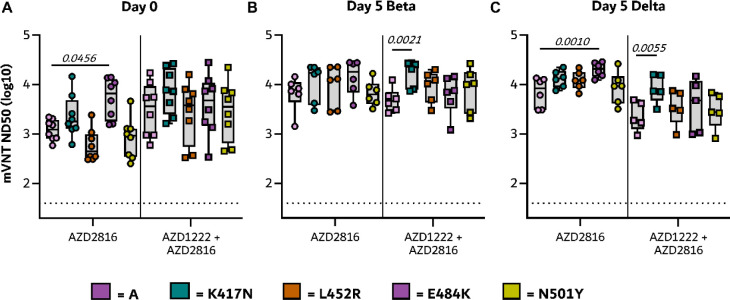
Humoral response of vaccinated hamsters against single mutant pseudotypes. Boxplots (minimum to maximum) of binding antibody titers as measured by pseudovirus VN titers in hamster sera obtained on day 0 (left panel), day 5 after Beta VoC challenge (middle panel), and day 5 after Delta VoC challenge (right panel). Statistical significance was determined via a Friedman test followed by Dunn’s multiple comparisons test comparing ancestral against mutant, p-values in italic when significant. N=6 per group, day 5 Delta prime boost group N=5.

**Figure 1.**
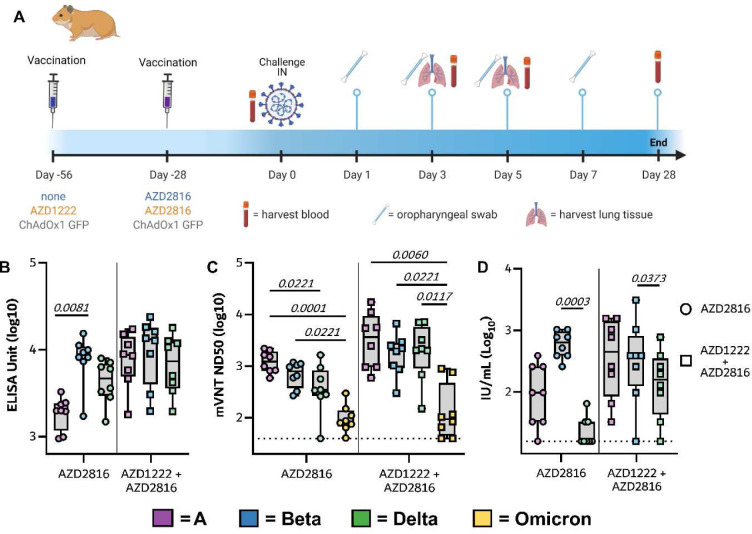
Humoral response of Syrian hamsters vaccinated with a single dose of AZD2816, a prime dose of AZD1222 and boost dose of AZD2816, or a prime-boost regimen of ChAdOx1 GFP.

We vaccinated Syrian hamsters either with a single dose of AZD2816 (prime only group, day -28, 2.5 × 10^8^ IU/animal, intramuscular injection), or with a prime dose of AZD1222 followed by a boost dose of AZD2816 (prime-boost group, day -56 and -28, both 2.5 × 10^8^ IU/animal, intramuscular injection), or with two injections of ChAdOx1 GFP (control group, day -56 and -28, 2.5 × 10^8^ IU/animal, intramuscular injection) (Figure 1A). On day 0, serum was obtained from eight hamsters per group and binding antibody titers against S protein were determined. In the prime-boost group, high binding titers were detected against all three S proteins. In the prime only group, binding antibody titers were significantly higher against Beta S compared to ancestral S (Figure 1B). Virus neutralizing (VN) antibody titers were determined using both a lentivirus-based pseudotype and live VN assay. Pseudotype VN titers were significantly lower against Omicron in both vaccine groups. In the prime only group, pseudotype VN titers were significantly lower for Delta than ancestral S (Figure 1C). Using the pseudotype VN assay, the influence of single substitutions K417N, N501Y (present in the Beta and Omicron VoC), E484K (present in the Beta VoC) as well as L452R (present in the Delta VoC) compared to ancestral spike were investigated. Significantly higher VN titers were found against the E484K mutant compared to ancestral S in prime only sera (Extended Data Figure 1A). Live VN titers were significantly higher against the Beta VoC compared to the Delta VoC in both vaccinated groups (Figure 1D). Together, these data show that a single vaccination with AZD2816 induces a robust immune response against S protein in hamsters, specifically against the Beta S or an S with the E484K mutation. However, vaccination with AZD1222 followed by AZD2816 does not result in a significant increase in binding to Beta or E484K S compared to ancestral S.

**Figure 2.**
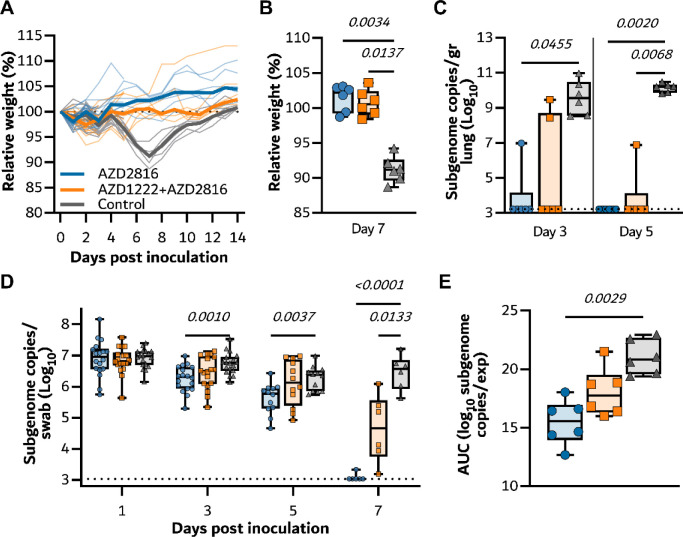
Vaccination of Syrian hamsters with AZD2816 or AZD1222 followed by AZD2816 reduces lower respiratory tract infection by the Beta VoC.

On Day 0, animals were challenged via the intranasal route with 10^4^ TCID_50_ of SARS-CoV-2 Beta VoC. Controls showed weight loss starting at day 5 and full recovery at day 12–14. In contrast, vaccinated hamsters maintained weight during the experiment (Figure 2A). Significant differences were observed between relative weights of the control and vaccinated groups on the day of peak weight loss compared to day 0 (Day 7, Figure 2B, p= 0.0034 and 0.0137, Kruskall Wallis test). On day 3 and 5, six animals per group were euthanized and lung tissue was collected. In lung tissue of controls, subgenomic viral RNA (sgRNA) was high on both days (12/12 samples positive, median of 3.6 × 10^9^ and 1.6 × 10^10^ copies/gram, respectively). In contrast, no or limited sgRNA was detected in lung tissue collected from prime only animals (1/6 and 0/6 samples positive on day 3 and 5 respectively, significant by Kruskall Wallis test). Likewise, sgRNA in lung tissue collected on day 5 from prime-boost animals was low (1/6 samples positive, significant by Kruskall Wallis test), whereas 2/6 samples collected on day 3 from the prime-boost group were found to be positive at levels equivalent to the control group (no significance, Figure 2C). Oropharyngeal swabs were collected on day 1, 3, 5, and 7, and the presence of sgRNA was investigated. No reduction of sgRNA load in swabs was detected on day 1 in vaccinated groups compared to the control. However, a significant reduction in sgRNA load was found on day 3 and 5 in prime only animals. On day 7, both vaccinated groups showed a reduction in sgRNA load in oropharyngeal swabs, showing that although vaccination did not prevent infection, it did significantly reduce the window of shedding (Figure 2D). Area-under-the-curve analysis, used as a measurement of total amount of sgRNA detected in oropharyngeal swabs throughout the experiment, showed a significant reduction in the prime only group, but not the prime-boost group, compared to controls (Figure 2E).

**Extended Data Figure 2.**
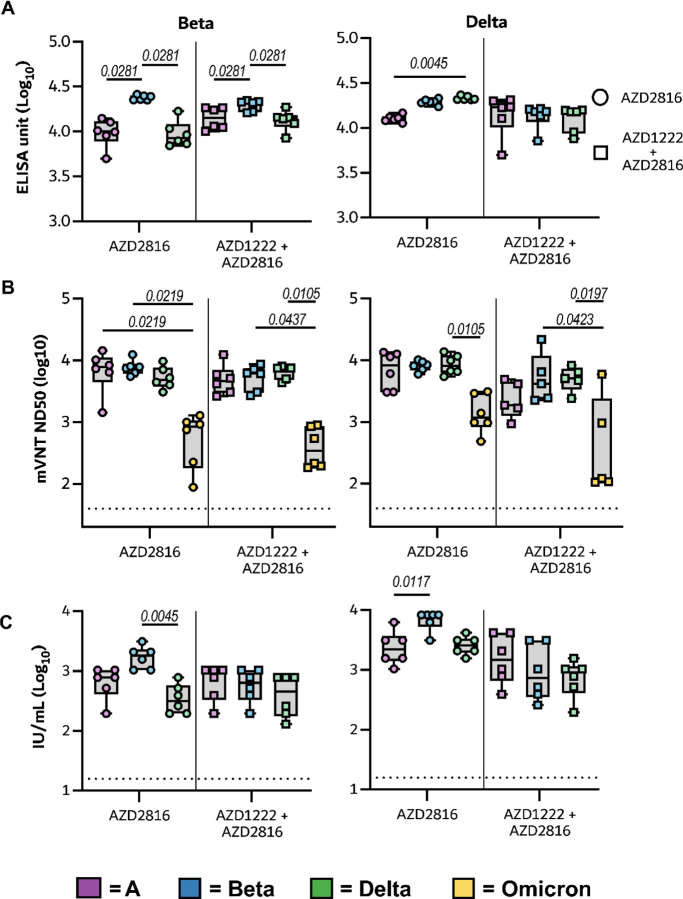
Humoral response of vaccinated hamsters upon challenge with the Beta (left panels) or Delta (right panels) VoC.

**Extended Data Figure 3.**
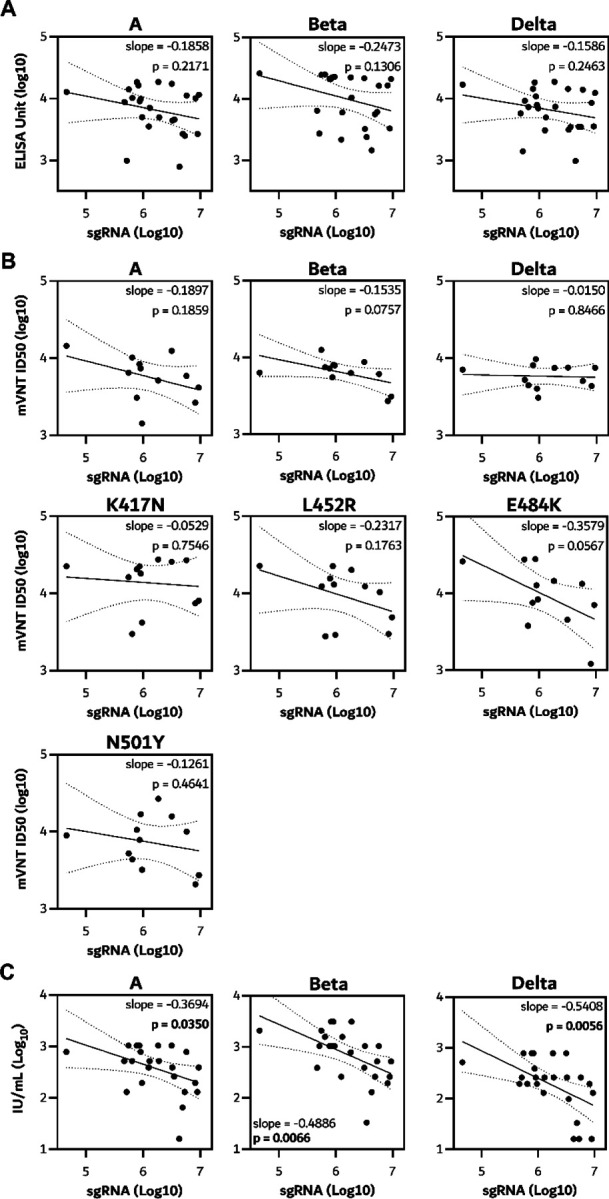
Linear correlation plots between sgRNA load in oropharyngeal swabs and antibodies found in serum at day of necropsy post Beta challenge.

Serum was collected from all hamsters on day 5 post challenge, and antibody titers were determined via ELISA as well as pseudovirus and live VN assays as described above (Extended Data Figure 2). A significantly higher binding antibody titer against Beta S compared to ancestral and Delta S was detected in both vaccine groups via ELISA. In live VN assays, higher VN titers were found against Beta compared to Delta VoC in the prime only group. In the pseudotype VN assay, titers against Omicron were lower than against ancestral, Beta and Delta. Furthermore, titers against K417N S were higher than ancestral S. (Extended Data Figure 1B and 2). We then investigated the correlation between sgRNA load in swabs on the day of necropsy with corresponding antibody levels. A significant correlation could be found between infectious VN titer against ancestral virus, the Beta, and Delta VoC, and sgRNA in swabs, but not between binding antibodies or pseudotype VN titer against any S variant and sgRNA in swabs (Extended Data Figure 3).

**Extended Data Figure 4.**
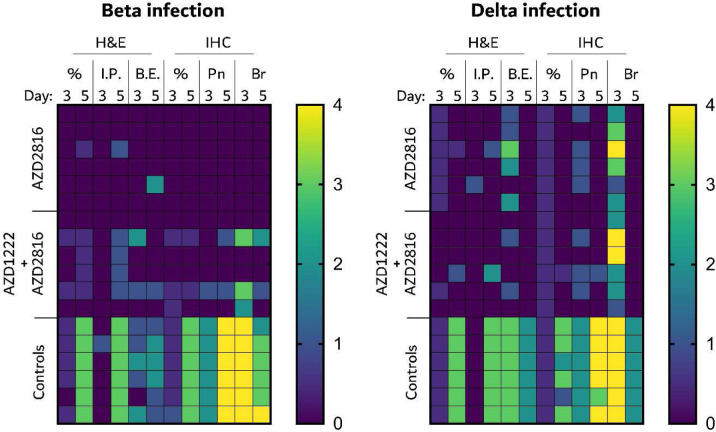
Heatmap of scores of pathological features in lung tissue of hamsters infected with the Beta or Delta variant. Each square represents an individual score, each column represents a pathological feature at either day 3 or day 5. All features were scored 0 to 5. H&E = hematoxylin and eosin stain. IHC = Immunohistochemistry for SARS2 antigen. % = percentage affected. I.P. = interstitial pneumonia. B.E. = Bronchiolitis with epithelial cell necrosis. Pn = Staining of type I and type II pneumocytes. Br = Staining of bronchiolar epithelium.

**Figure 3.**
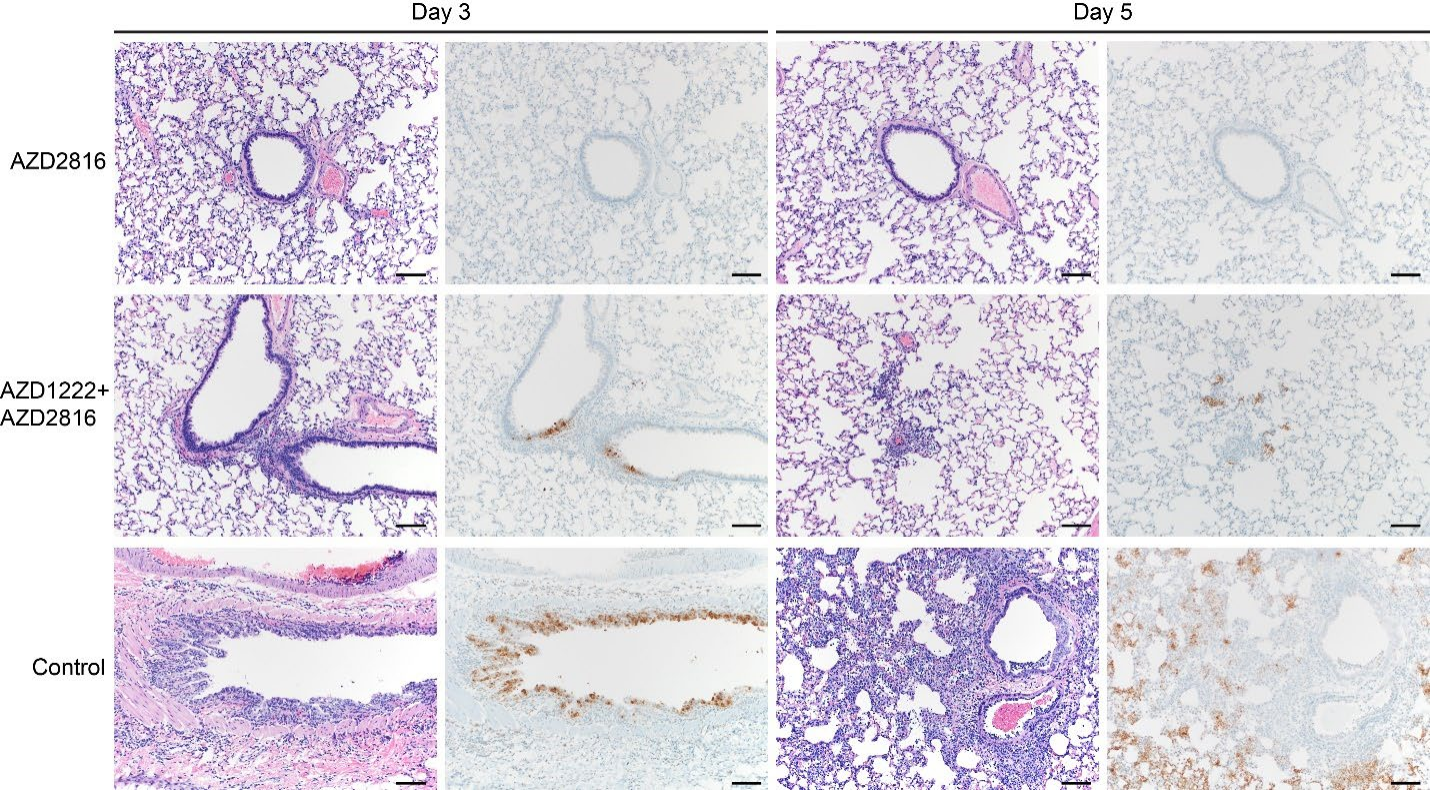
Pulmonary effects of intranasal challenge with the Beta VoC in vaccinated and control hamsters at day 3 and 5 post challenge. H&E staining (1^st^ and 3^rd^ column) and IHC staining against N protein (brown, 2^nd^ and 4^th^ column), 100x, scale bar = 100 μm. No pathology nor antigen staining observed in animals which received an AZD2816 vaccination. No pathology observed in animals which received an AZD1222 + AZD2816 vaccination. Compared to control, limited staining of bronchiolar epithelium observed on day 3 and 5. Control animals show progression from bronchiolitis on day 3 to bronchointerstitial pneumonia on day 5, at which point alveolar septa are expanded by edema fluid and leucocytes. Staining of bronchiolar epithelial cells, type I&II pneumocytes, and rare macrophages. Images are representative of observations within 100% of a complete lung section containing all lobes.

Lung pathology was scored by a board-certified veterinary pathologist blinded to study groups (Figure 3). SARS-CoV-2-related pathology was observed in all animals of the control group. On day 3 post Beta challenge, minimal-to-moderate acute bronchiolitis was observed affecting less than 1% of the lung. Histological lesions consisted of a moderate subacute broncho-interstitial pneumonia affecting between 30–50% of pulmonary tissue. Lesions were characterized by broncho-interstitial pneumonia centered on terminal bronchioles and extending into the adjacent alveoli. Alveolar septa were expanded by edema fluid and leucocytes. SARS-CoV-2 antigen staining was numerous within bronchiolar epithelium on day 3, whereas this had mostly moved to type I and II pneumocytes on day 5. In contrast, antigen staining in the vaccinated groups was relatively low; samples obtained from the prime only group were mostly negative, whereas antigen staining in samples obtained from the prime-boost group was between none to moderate (Figure 3, Extended Data Figure 4).

**Figure 4.**
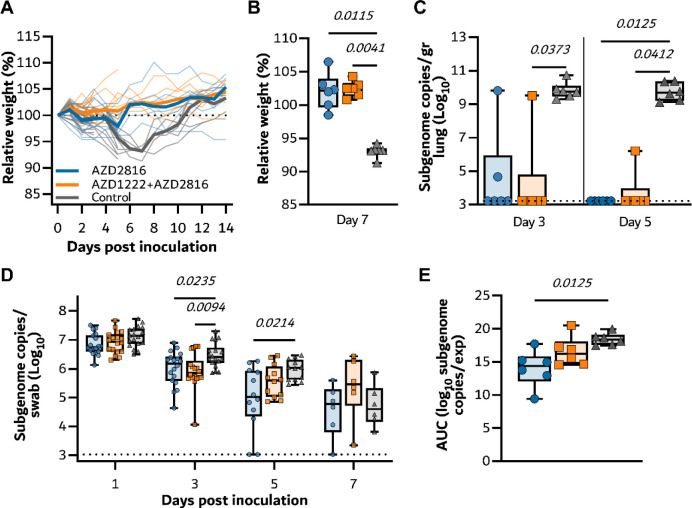
Vaccination of Syrian hamsters with AZD2816 or AZD1222 followed by AZD2816 reduces lower respiratory tract infection by the Delta VoC.

To investigate the efficacy of a vaccine optimized for the Beta S against the Delta variant, new groups of hamsters vaccinated as described above were challenged with 10^4^ TCID_50_ of the Delta variant via the intranasal route. As observed upon challenge with the Beta variant, vaccinated animals did not show any weight loss throughout the experiment, whereas control animals did lose weight (Figure 4A). Indeed, differences in weight loss between the control and vaccinated groups were significant on day 7 (Figure 4B). High levels of sgRNA could be detected on both day 3 and 5 in lung tissue of control animals (12/12 samples positive, median of 5.6 × 10^9^ and 5.0 × 10^9^ copies/gram, respectively). In contrast, the majority of lung tissue obtained from vaccinated animals was negative for sgRNA, viral sgRNA was detected in 1/6 samples on each day for the prime boost group, versus 2/6 on day 3 and 0/6 on day 5 for the prime only group (Figure 4C). Significant differences in sgRNA detected in oropharyngeal swabs were limited to day 3 (both vaccinated groups) and day 5 (prime only group) compared to controls (Figure 4D). In contrast to what we observed in animals inoculated with the Beta variant, we did not see a decrease in the window of shedding of vaccinated animals compared to control animals. Area-under-the-curve analysis (as a measurement of total amount of sgRNA detected in oropharyngeal swabs throughout the experiment) showed that animals that received a prime only vaccination shed significantly less than control animals (Figure 4E).

**Extended Data Figure 5.**
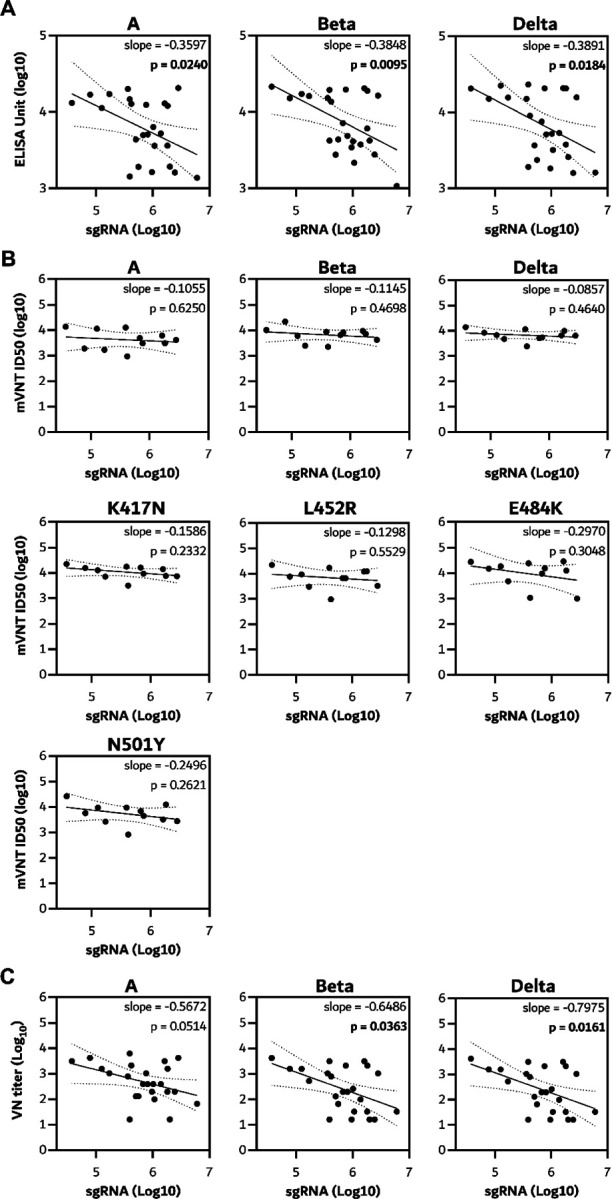
Linear correlation plots between sgRNA load in oropharyngeal swabs and antibodies found in serum at day of necropsy post Delta challenge.

In sera collected on day 5 post challenge, a significantly higher binding antibody titer against Delta S compared to ancestral S was detected in the prime only group (Extended Data Figure 2). In the live VN assay, higher VN titers were found against the Beta VoC compared to ancestral virus in the prime only group (Extended Data Figure 2). Significant differences in the pseudotype VN assay were found between Omicron and Beta as well as Delta VoCs (Extended Data Figure 2), as well as between ancestral and E484K S in the prime only group, and ancestral and K417N S in the prime boost group. (Extended Data Figure 1C). A linear correlation was found between sgRNA in swabs and correlating binding antibodies against ancestral, Beta and Delta S. For live VN titers, a significant correlation was found with the Beta and Delta, but not ancestral, VoCs (Extended Data Figure 5).

**Figure 5.**
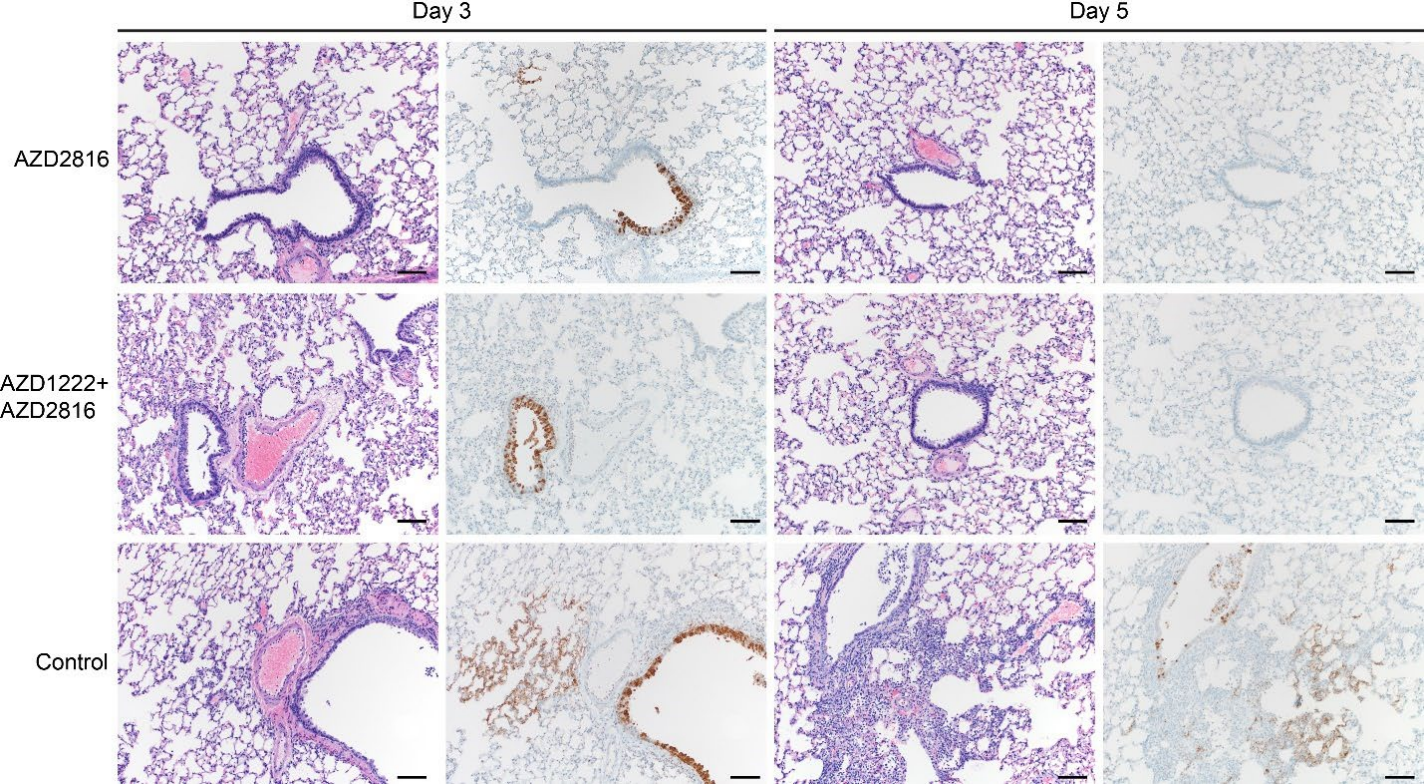
Pulmonary effects of intranasal challenge with the Delta VoC in vaccinated and control hamsters at day 3 and 5 post challenge. H&E staining (1^st^ and 3^rd^ column) and IHC staining against N protein (brown, 2^nd^ and 4^th^ column), 100x, scale bar = 100 μm; Limited bronchiolitis with epithelial cell necrosis observed on day 3, which was resolved on day 5, in animals that received an AZD2816 vaccination. No pathology observed in animals which received an AZD1222 + AZD2816 vaccination. Compared to controls, limited staining of bronchiolar epithelium observed on day 3, which was resolved on day 5 in both vaccine groups. Control animals show progression from bronchiolitis on day 3 to bronchointerstitial pneumonia on day 5, at which point alveolar septa are expanded by edema fluid and leucocytes. Staining of bronchiolar epithelial cells, type I&II pneumocytes, and rare macrophages on both days. Images are representative of observations within 100% of a complete lung section containing all lobes.

SARS-CoV-2 -related pathology post Delta challenge was observed in all animals of the control group and did not differ from animals challenged with the Beta VoC (Figure 5, Extended Data Figure 4). In the prime only group, a minimal-to-moderate bronchiolitis was observed in some animals on day 3. In the prime boost group, bronchiolitis was either absent or minimal. This was combined with reduced antigen staining in the bronchiolar epithelium in both groups compared to controls. No pathology or antigen staining was observed on day 5, except for one animal in the prime boost group, which is the only animal that was positive for sgRNA at this time point.

**Figure 6.**
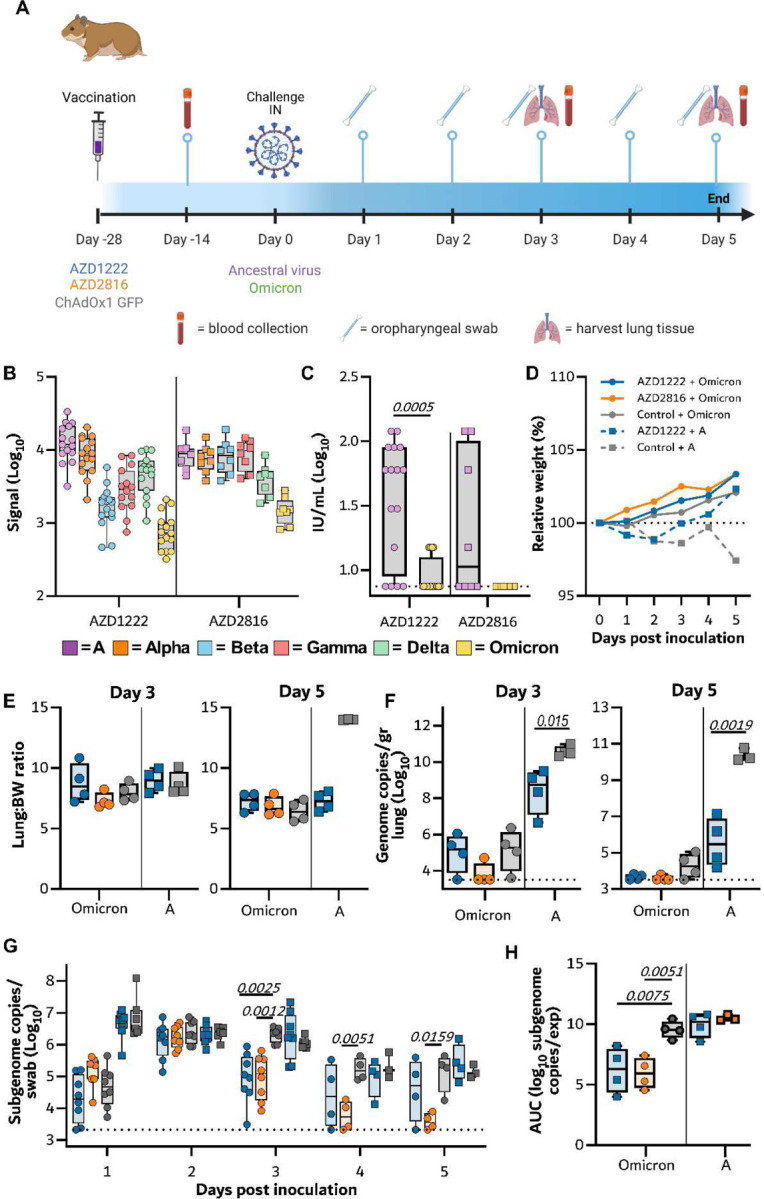
Vaccination of Syrian hamsters with AZD2816 or AZD1222 reduces shedding by the Omicron VoC. A) Schematic overview of experiment. Hamsters were vaccinated with AZD1222, AZD2816, or ChAdOx1 GFP on day –28. Twenty-eight days post final vaccination, hamsters were challenged with 10^3^ TCID_50_ of the Omicron or ancestral variant, via the intranasal route. B) Boxplot (minimum to maximum) of binding IgG antibody signal in hamster sera obtained on day -14 against different SARS-CoV-2 S proteins obtained using the V-PLEX SARS-CoV-2 panel 23 by Meso Scale Discovery. Circles = hamsters vaccinated with AZD2816, squares = hamsters vaccinated with AZD2816. C) Boxplot (minimum to maximum) of virus neutralizing antibody titers in hamster sera obtained on day -14 against different ancestral virus or Omicron VoC. VN titers were normalized against NIBSC standard. Statistical significance was determined via a Wilcoxon test. Circles = hamsters vaccinated with AZD2816, squares = hamsters vaccinated with AZD2816. D) Relative weight in comparison to day 0. Dotted line = 100% relative weight. N=8 (Day 1–3) or 4 (Day 4–5). Circles = hamsters challenged with Omicron, squares = hamsters challenged with ancestral variant. E) Boxplot (minimum to maximum) of lung:body weight ratio. Circles = hamsters challenged with Omicron, squares = hamsters challenged with ancestral variant. F) Boxplot (minimum to maximum) of gRNA in lung tissue harvested on day 3 and 5 (N=4). Statistical significance was determined via a Kruskall Wallis test followed by Dunn’s multiple comparisons test. Dotted line = limit of detection. Circles = hamsters challenged with Omicron, squares = hamsters challenged with ancestral variant. G) Boxplot (minimum to maximum) of sgRNA in oropharyngeal swabs taken on day 1–3 (N=8), and 4–5 (N=4). Statistical significance was determined via a mixed-effects analysis followed by Dunnett’s multiple comparisons test, comparing vaccinated groups against control group. Dotted line = limit of detection. Circles = hamsters challenged with Omicron, squares = hamsters challenged with ancestral variant. H) Boxplot (minimum to maximum) of the AUC analysis of shedding as measured by sgRNA analysis in swabs collected on 1–5 days post inoculation. Circles = hamsters challenged with Omicron, squares = hamsters challenged with ancestral variant. Statistical significance was determined via one-way ANOVA. N=5.

We then investigated the efficacy of AZD1222 and AZD2816 against the Omicron VoC. Here, groups of hamsters were vaccinated with a single dose of AZD1222, AZD2816, or ChAdOx1 GFP and serum was collected 14 days post-vaccination (Figure 6A). Binding antibodies against different S proteins were detected using the Mesoscale V-PLEX SARS-CoV-2 panel 23, in-house optimized for hamster sera. Upon vaccination with AZD1222, binding antibodies were highest for ancestral and Alpha S and lowest for Omicron. Upon vaccination with AZD2816, antibody levels were similar for ancestral, Alpha, Beta, and Gamma S, but dropped for Delta and Omicron S (Figure 6B). Live VN titers against the Omicron VoC were significantly lower compared to the ancestral variant (Figure 6C). 28 days after vaccination, animals were challenged with the ancestral variant or Omicron VoC. As previously reported^25^, we did not see weight loss in control hamsters challenged with the Omicron VoC, whereas this weight loss was present in control hamsters challenged with ancestral virus (Figure 6D). Four animals per group were euthanized on day 3 and day 5. No significant differences in lung:body weight ratio were observed, although lung:body weight ratio was relatively high on day 5 for control hamsters inoculated with ancestral virus (Figure 6E). As expected, AZD1222 vaccination resulted in significantly reduced viral genome copies in lung tissue (Figure 6F). However, replication of the Omicron VoC in hamster lung tissue was low and although a reduction in genome copies in lung tissue of vaccinated hamsters was observed, particularly in the animals which received AZD2816, no significance was reached.

Oropharyngeal swabs were obtained on day 1–5 and analyzed for sgRNA. Shedding of the control animals infected with the Omicron VoC was similar to control and vaccinated animals that were infected with the ancestral variant, albeit lower on day 1. However, vaccinated animals inoculated with the Omicron VoC had significantly lower shedding on day 3 (both groups), day 4, and day 5 (AZD2816 vaccinated group only) compared to controls (Figure 6G). Area-under-the-curve analysis was performed on the four animals per group that were euthanized on day 5 as a measure of total amount of virus shed throughout the experiment. Significantly lower shedding was detected in the vaccinated animals infected with the Omicron VoC, but not in those infected with the ancestral virus (Figure 6H).

**Extended Data Figure 6.**
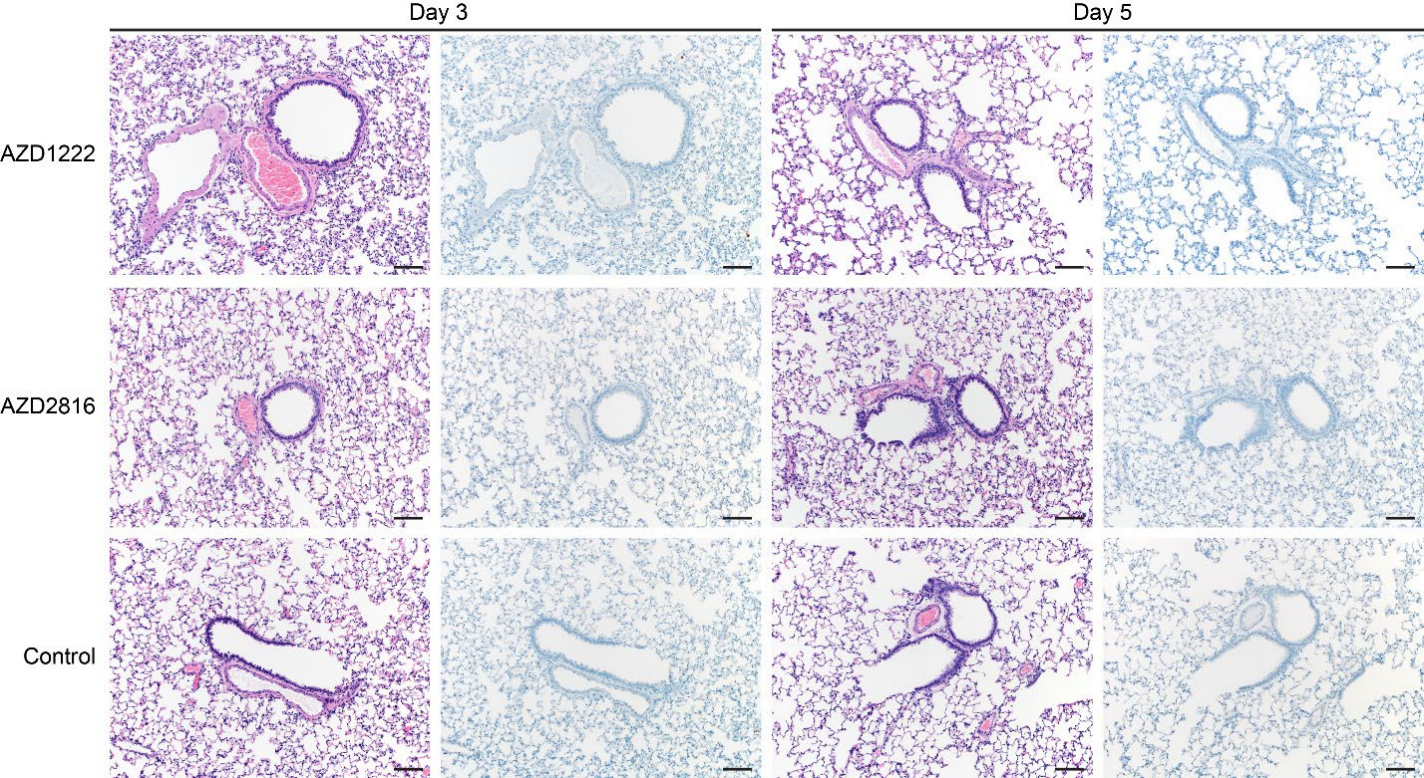
Pulmonary effects of intranasal challenge with the Omicron VoC in vaccinated and control hamsters at day 3 and 5. H&E staining (1st and 3rd column) and IHC staining against N protein (brown, 2nd and 4th column), 100x, scale bar = 100 μm; Most vaccinated and control animals showed no pathology in the lower respiratory tract, except for minimal interstitial pneumonia on day 5 in 1/4 animals in each vaccine group. Antigen staining was limited to bronchial and bronchiolar epithelium in 2/4 control animals on day 5 and 1/4 AZD1222 vaccinated animals on day 3, as well as in type I and II pneumocytes in l/4 animals in control animals on day 5.

**Figure 7.**
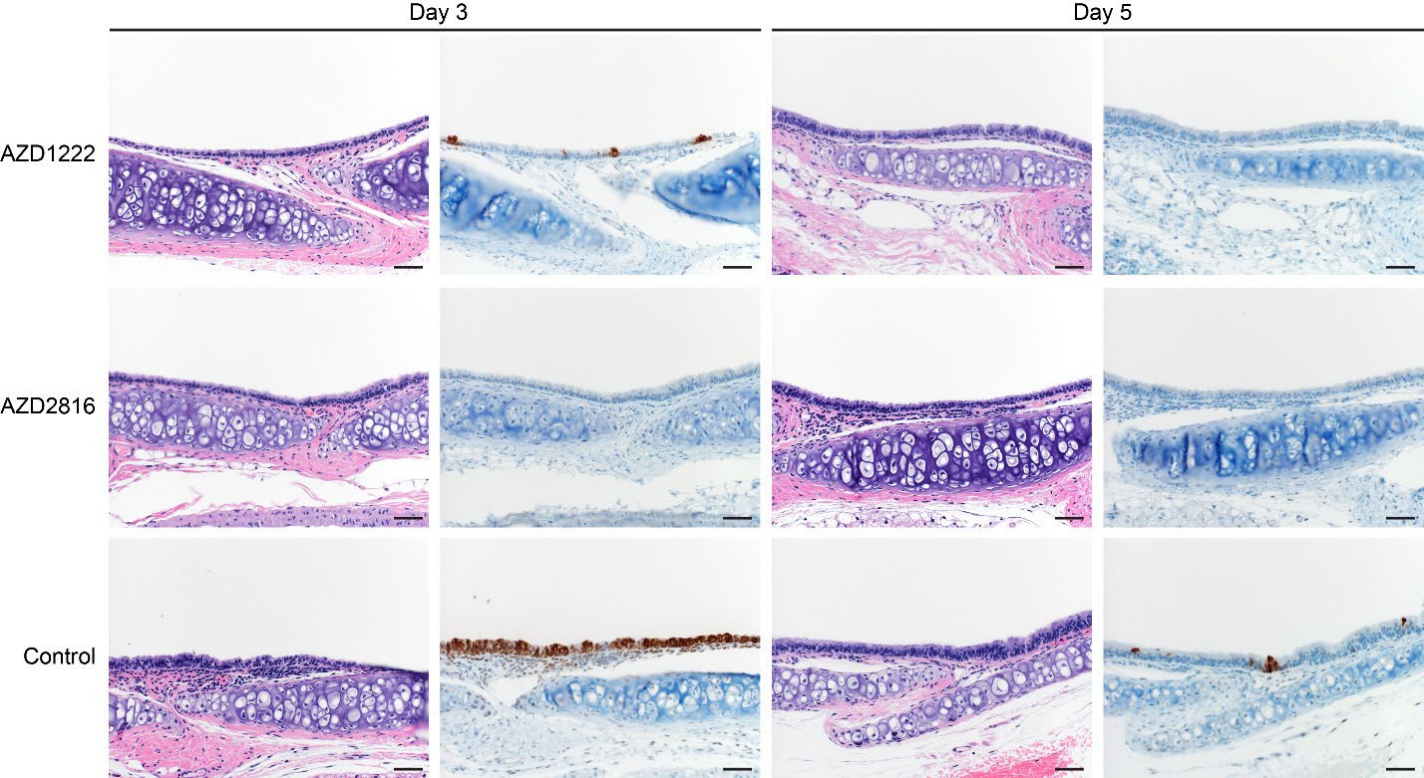
Effects of intranasal challenge with the Omicron VoC on tracheal tissue in vaccinated and control hamsters at day 3 and 5 post challenge.

Lung pathology was scored by a board-certified veterinary pathologist blinded to study groups (Figure 7, Extended Data Figure 6). SARS-CoV-2-related pathology differed from what was observed in the animals inoculated with the Beta or Delta VoC. On day 3 and 5 post Omicron challenge, no evidence of pulmonary pathology was noted in the lower airway in any of the control animals, however minimal subacute inflammation and multifocal necrosis was noted in the trachea of 2/4 and 1/4 animals on day 3 and 5, respectively. SARS-CoV-2 antigen staining was observed in 3/4 tracheal tissues on day 3. On day 5, rare staining was observed in bronchial and bronchiolar epithelium in 2/4 animals, in type I and II pneumocytes in l/4 animals, and in tracheal epithelium in 1/4 animals. In animals vaccinated with AZD1222, 1/4 animals had a minimal subacute inflammation and multifocal necrosis of the tracheal epithelium. Rare SARS-CoV-2 antigen staining was observed in tracheal epithelium of 3/4 animals, and scattered SARS-CoV-2 antigen staining was observed in bronchial and bronchiolar epithelium of 1/4 animals. On day 5, no SARS-CoV-2 antigen staining is observed, and minimal interstitial pneumonia was seen in 1/4 animals. In animals vaccinated with AZD2816, no pathology was observed on day 3, and limited to minimal interstitial pneumonia in 1/4 animals on day 5. Antigen staining was limited to rare tracheal epithelium staining on day 3, with no staining observed on day 5 (Figure 7, Extended Data Figure 6).

## Discussion

We have previously shown that despite reduced neutralizing antibody titers in sera obtained from hamsters vaccinated with AZD1222 against the Beta VoC, hamsters were fully protected against lower respiratory tract infection^26^. Other vaccines have performed differently in animal models. Tostanoski *et al*. show that although vaccination with Ad26.COV2.S (an adenovirus-vectored vaccine encoding ancestral S protein) reduced the viral load detected in lung tissue of hamsters challenged with the Beta VoC at 14 days post challenge, this difference was not significant for gRNA^27^. Likewise, rhesus macaques vaccinated with the same vaccine showed higher viral loads in bronchoalveolar lavage and nasal swabs when challenged with the Beta VoC compared to ancestral SARS-CoV-2^28^. Finally, Corbett *et al*. show that sgRNA was detected in bronchoalveolar lavage and nasal swabs from rhesus macaques vaccinated with the Moderna vaccine mRNA-1273 (encoding ancestral S) and challenged with the Beta VoC^29^, whereas this was limited in rhesus macaques that were challenged with ancestral virus^30^. These data suggest that while vaccines which encode the ancestral spike can protect against hospitalization and death caused by VoC, a variant-specific vaccine may result in increased protection against disease and onward transmission.

Thus, we investigated the protective efficacy of the vaccine AZD2816, which encodes the S protein of the Beta VoC, in the hamster model. In contrast to control animals, upon challenge with either the Beta, Delta, or Omicron VoC, little-to-no viral RNA was found at 5 days post challenge in the lower respiratory tract of the vaccinated hamsters. Thus, the vaccine regimens utilized in the current study, including single dose AZD2816, are protective against all three VoCs in the hamster model.

Vaccine and variant-specific differences were observed in the different experiments. In the Beta VoC study, lung tissue from 2/6 prime boost vaccinated animals were positive for sgRNA at day 3, combined with higher antigen staining in this group compared to the prime only group. Furthermore, whereas total shedding was reduced in the prime only group compared to controls, this was not the case for the prime boost group. This suggests that initial priming with one VoC S may shape the immune response to subsequent vaccinations. Indeed, our humoral immune response analysis showed higher titers for the Beta S and E484K mutation compared to ancestral or Delta S in the prime only group, but not the prime boost group. In contrast, in the Delta VoC study, the prime boost group appeared to be slightly better protected than the prime only group, mostly evident in pathology scoring. This may be due to the higher quantity of antibodies in the prime boost group compared to the prime only group.

Similar to what has been reported by other groups, replication of the Omicron VoC was limited in the lower respiratory tract^25,31^. We were unable to detect any sgRNA, and gRNA was low compared to ancestral virus. Despite the limited replication in the lower respiratory tract, the upper respiratory tract displayed much higher viral replication, comparable to ancestral virus. Both the AZD1222 and AZD2816 vaccine were able to reduce shedding within the Omicron-challenged groups suggesting that both vaccines are effective. This was recently confirmed in a preprint by Gagne *et al*., in which they show protection of NHPs against the Omicron VoC, both with a regimen of mRNA-1273 against ancestral S, 3x, or mRNA-1273 2x and mRNA-Omicron as a booster^32^. Further research is required to determine to the extent in which the hamster model recapitulates human disease and infection kinetics with the Omicron VoC. For example, a higher inoculum dose as well as a different inoculation route such as intratracheal may increase virus replication in the lower respiratory tract.

Interestingly, whereas we previously reported on the lack of reduction in virus detected in oropharyngeal swabs when vaccines were given via the intramuscular route^33,34^, vaccinated hamsters inoculated with the Omicron VoC displayed significantly reduced shedding compared to controls, even though shedding of control animals was at levels equal to animals inoculated with the ancestral virus. This difference was particularly evident in the AZD2816 group. Omicron has an E484A mutation in the S protein, whereas the Beta VoC has an E484K mutation. In our pseudovirus VN assays, we show a higher neutralization of pseudotypes with the E484K mutation compared to ancestral S in serum obtained from hamsters that only received the AZD2816 vaccination. It is possible that this also translates to the E484A mutation.

However, we did not see an increase neutralization in live virus assays against the Omicron VoC in serum obtained from hamsters vaccinated with AZD2816 compared to those vaccinated with AZD1222. Further research is needed to determine whether the small difference observed between the two vaccines against the Omicron VoC is relevant, and why shedding is reduced.

A significant correlation was found between sgRNA load in oropharyngeal swabs and neutralizing antibody titers in animals, whereas binding antibodies titers were also predictors for sgRNA load in swabs in animals challenged with the Delta VoC. This finding confirms previous reports of a correlation between binding and neutralizing antibodies and viral load in both hamsters^35^ and non-human primates^29,36^.

Our study confirms that AZD2816 is immunogenic in the hamster model and protects against infection of the lower respiratory tract against the Omicron, Beta, and Delta VoC. Likewise, a single dose of AZD1222 protects against the Omicron VoC. Furthermore, initial immunization with AZD1222 followed by immunization with AZD2816 results in full protection against the Beta and Delta VoCs, and we predict it will also protect against Omicron. This confirms previous reports that a full antigenic match between the vaccine and the challenged virus is not required for protection of the lower respiratory tract.

## Materials and Methods

### Ethics Statement

Animal experiments were conducted in an AAALAC International-accredited facility and were approved by the Rocky Mountain Laboratories Institutional Care and Use Committee following the guidelines put forth in the Guide for the Care and Use of Laboratory Animals 8^th^ edition, the Animal Welfare Act, United States Department of Agriculture and the United States Public Health Service Policy on the Humane Care and Use of Laboratory Animals.

The Institutional Biosafety Committee (IBC) approved work with infectious SARS-CoV-2 virus strains under BSL3 conditions. Virus inactivation of all samples was performed according to IBC-approved standard operating procedures for the removal of specimens from high containment areas.

### Cells and virus

SARS-CoV-2 variant B.1.351 (USA/MD-HP01542/2021, EPI_ISL_890360) was obtained from Andrew Pekosz at John Hopkins Bloomberg School of Public Health. SARS-CoV-2 variant B.1.617.2 (hCoV-19/USA/KY-CDC-2-4242084/2021) was obtained from BEI resources. SARS-CoV-2 variant B.1.1.529 (hCoV-19/USA/GA-EHC-2811C/2021, EPI_ISL_7171744) was obtained from Mehul Suthar, Emory University. All virus stocks were sequenced, and no SNPs compared to the patient sample sequence were detected. Virus propagation was performed in VeroE6 cells in DMEM supplemented with 2% fetal bovine serum, 1 mM L-glutamine, 50 U/ml penicillin and 50 μg/ml streptomycin (DMEM2). VeroE6 cells were maintained in DMEM supplemented with 10% fetal bovine serum, 1 mM L-glutamine, 50 U/ml penicillin, and 50 μg/ml streptomycin. No mycoplasma was detected in cells or virus stocks.

### Animal Experiments

ChAdOx1 nCoV-19 was formulated as previously described^37^. 4–6-week-old Syrian hamsters (Envigo Indianapolis) were vaccinated with 2.5 × 10^8^ infectious units of AZD1222, AZD2816, or ChAdOx1-GFP delivered intramuscularly in two 100 μL doses into the posterior thighs 56 or 28 days prior to challenge. Prior to challenge, a serum sample was collected via the retro-orbital plexus under isoflurane anesthesia. All animals were challenged intranasally with 40 μl containing 10^4^ TCID_50_/mL virus in sterile DMEM. Body weights were recorded daily. Oropharyngeal swabs were collected in 1 mL of DMEM2. On day 3 and 5, 4–6 animals from each group were euthanized and lung samples were taken for qRT-PCR analysis, virus titrations and histopathology. The remaining six animals in each group were monitored daily until day 21.

### RNA extraction and quantitative reverse-transcription polymerase chain reaction

RNA was extracted from DMEM2 containing oropharyngeal swabs using the QiaAmp Viral RNA kit (Qiagen), and lung samples were homogenized and extracted using the RNeasy kit (Qiagen) according to the manufacturer’s instructions and following high-containment laboratory protocols. Five μL of extracted RNA was tested with the Quantstudio 3 system (Thermofisher) according to the manufacturer’s instructions using viral RNA specific assays^38,39^. A standard curve was generated during each run using SARS-CoV-2 standards containing a known number of genome copies.

### Virus neutralization

Sera were heat-inactivated (30 min, 56 °C). After an initial 1:10 dilution of the sera, two-fold serial dilutions were prepared in DMEM2. 100 TCID_50_ of SARS-CoV-2 was added to the diluted sera. After a 60 min incubation at 37°C and 5% CO_2_, the virus-serum mixture was added to VeroE6 cells and cells were further incubated for 6 days before assessment of CPE. The virus neutralization titer was expressed as the reciprocal value of the highest dilution of the serum that still inhibited virus replication. Three different positive serum controls were done next to NIBSC sera sample 20/130 by three different technicians, to determine IU/mL equivalent. NIBSC sera readout was 640–1066, compared to reported value at 1300 (1.5x higher). All serum samples were subsequently accompanied by positive controls on the plate. Assays were only approved if positive controls fell within the range previously determined by three technicians. Values were then multiplied by 1.5 to determine IU/mL.

### Generating lentiviral based pseudotypes bearing the SARS-CoV-2 S protein

Lentiviral-based SARS-CoV-2 pseudotyped viruses were generated in HEK293T cells incubated at 37°C, 5% CO2 as previously described^40^. Mutant SARS-CoV-2 expression plasmids (Clade A, Beta, Delta, Omicron, N501Y, E484K, K417N, L452R) were generated by site-directed mutagenesis or using the QuikChange Lightning Multi Site-Directed Mutagenesis Kit (Agilent). All SARS-CoV-2 spike expression plasmids were based on the Wuhan-hu-1 reference sequence^41^, with the additional substitutions D614G (except for clade A) and K1255*STOP (aka the Δ19 mutation or cytoplasmic tail truncation). Briefly, HEK293T cells were transfected with SARS-CoV-2 spike, along with the lentiviral plasmids p8.91 (encoding for HIV-1 gag-pol) and CSFLW (lentivirus backbone expressing a firefly luciferase reporter gene) with PEI (1 μg/mL) transfection reagent. Supernatants containing pseudotyped SARS-CoV-2 were harvested and pooled at 48 and 72 hours post transfection, centrifuged at 1,300 × g for 10 minutes at 4 °C to remove cellular debris and stored at −80 °C. SARS-CoV-2 pseudoparticles were titrated on HEK293T cells stably expressing human ACE2 and infectivity assessed by measuring luciferase luminescence after the addition of Bright-Glo luciferase reagent (Promega) and read on a GloMax Multi+ Detection System (Promega).

### Micro neutralization test (mVNT) using SARS-CoV-2 pseudoparticles

Sera was diluted 1:20 in serum-free media in a 96-well plate in triplicate and titrated 3-fold. A fixed volume of SARS-CoV-2 pseudoparticles were added at a dilution equivalent to 10^5^ signal luciferase units in 50 μL DMEM-10% and incubated with sera for 1 hour at 37 °C, 5% CO2 (giving a final sera dilution of 1:40). Target cells stably expressing human ACE2 were then added at a density of 2 × 10^4^ in 100 μL and incubated at 37 °C, 5% CO_2_ for 48 hours. Firefly luciferase activity was then measured after the addition of Bright-Glo luciferase reagent on a Glomax-Multi+ Detection System (Promega, Southampton, UK). CSV files were exported for analysis. Pseudotyped virus neutralization titers were calculated by interpolating the dilution at which a 50% reduction in reduction in luciferase activity was observed, relative to untreated controls, neutralization dose 50% (ND50).

### Enzyme-linked immunosorbent assay

MaxiSorp plates (Nunc) were coated with 100ng (2μg/ml) whole spike protein diluted in PBS for overnight adsorption at 4°C. Plates were washed in PBS/Tween (0.05% v/v) and wells blocked using casein (ThermoFisher Scientific) for at least 1 hr at RT. Standard positive sera (pool of hamster serum from AZD1222-AZD2816 vaccinated animals with high endpoint titer against original spike protein), individual hamster serum, negative and internal control samples were added to plates and incubated for at least 2 hours at RT. Following washing, bound antibodies were detected by addition of Alkaline Phosphatase-conjugated goat anti-hamster IgG (Sigma-Aldrich, SAB3700455) (1:1000 dilution) for 1hr at RT and addition of p-Nitrophenyl Phosphate, Disodium Salt substrate (Sigma-Aldrich) and optimal density reading at 405nm. An arbitrary number of ELISA units (EU) were assigned to the reference pool and optical density values of each dilution were fitted to a 4-parameter logistic curve using SOFTmax PRO software. ELISA units were calculated for each sample using the optical density values of the sample and the parameters of the standard curve. All data was log-transformed for presentation and statistical analyses.

### Binding antibody titers against different spike proteins on the Meso Quickplex

The V-PLEX SARS-CoV-2 Panel 13 (IgG) kit (MSD, K15463U) was used to run the hamster samples on the Meso Quickplex (MSD, K15203D). The 96-well plate was incubated with 150 μL of Blocker A solution at room temperature with shaking for 30 minutes, then washed 3 times with 150 μL/well of MSD Wash buffer. 50 μL of the standard curve and hamster samples were transferred to the plate in duplicates. Vaccinated hamster serum samples were diluted 10,000x, and ChAdOx1 GFP-vaccinated hamster serum samples were diluted 1,000x. The plate was sealed with shaking at room temperature for 2 hours, followed by 3 washes with 1X MSD Wash buffer. An in-house MSD GOLD SULFO-TAG NHS-Ester (MSD, R31AA-2) conjugated goat anti-hamster IgG secondary antibody (Thermo Fischer, SA5-10284) was diluted 10,000x in diluent 100 and 50 μL was applied to each well of the plate. The plate was sealed with shaking at room temperature for 1 hour. After incubation, the plate was washed with 1X MSD Wash buffer as before, and 150 μL of MSD Gold Read Buffer B was added per well. The plate was read immediately by the MSD instrument. Arbitrary units (AU) were assigned to the standard curve of pooled SARS-CoV-2-positive hamster sera, which was used on each plate. AU/mL were calculated using the MSD Workbench 4.0 software.

### Histology and immunohistochemistry

Lungs were perfused with 10% neutral-buffered formalin and fixed for at least 8 days. Tissue was embedded in paraffin, processed using a VIP 6 Tissue-Tek (Sakura Finetek) tissue processor, and then embedded in Ultraffin paraffin polymer (Cancer Diagnostics). Sections of 5 μm were deparaffinized in xylene, passed through 100% ethanol, and rehydrated in tap water. Sections were stained with Harris hematoxylin (Cancer Diagnostics, no. SH3777), decolorized with 0.125% HCl/70% ethanol, blued in Pureview PH Blue (Cancer Diagnostics, no. 167020), counterstained with eosin 615 (Cancer Diagnostics, no. 16601), dehydrated, and mounted in Micromount (Leica, no. 3801731). An in-house–generated SARS-CoV-2 nucleocapsid protein rabbit antibody (GenScript) at a 1:1000 dilution was used to detect specific anti–SARS-CoV-2 immunoreactivity, carried out on a Discovery ULTRA automated staining instrument (Roche Tissue Diagnostics) with a Discovery ChromoMap DAB (Ventana Medical Systems) kit. The tissue slides were examined by a board-certified veterinary anatomic pathologist blinded to study group allocations. Scoring was done as follows. H&E; no lesions = 0; less than 1% = 0.5; minimal (1–10%) = 1; mild (11–25%) = 2; moderate (26–50%) = 3; marked (51–75%) = 4; severe (76–100%) = 5. IHC attachment; none = 0; less than 1% = 0.5; rare/few (1–10%) = 1; scattered (11–25%) = 2; moderate (26–50%) = 3; numerous (51–75%) = 4; diffuse (76–100%) = 5.

### Data availability statement

Data will be deposited in Figshare.

## Extended Data
